# Effects of Intermarriage

**Published:** 1858

**Authors:** 


### Effects of Intermarriage.

Intermarriage among blood relations produces bad results. The editor of the Fredericksburg (Va.) News says:

"In this county, in which we are raised, for twenty generations back certain families of wealth and respectability have intermarried until there cannot be found, in three or four of them, a sound man or woman! One has sore eyes; another scrofula; a third is an idiot; a fourth blind; a fifth bandy-leged; a sixth with a head about the size of a turnip—with not one out of the number exempt from physical defects of some kind or other."

We witnessed an example of the deteriorating effects of intermarriage in an old settled county in an Eastern State, into which, we believe, an immigrant was not seen for a century and upward. In one locality the entire population, almost, were tied together remotely or more nearly by consanguinity and the result was but too apparent in the imperfectly developed forms and feeble intellects. In one family the father was the offspring of first cousins, and so, also, was the mother the product of the union of cousins. The children of this father and mother (six in number) each wore upon his and her hand, four fingers, and upon the feet six toes. The hair was silky white upon the heads of all, the skin was chalky white, the eyes were pale blue, the conjunction red, with vessels and the mucus lining of the cilia intensely red colored, giving them somewhat the appearance of Albinos. They were low in stature and thick set; and when standing out in the light of the sun their brows were knit and the cilia brought down to protect the eyes from the light. Such in part their physical organization, but their mental or intellectual, was even worse. Imbecility was marked upon the countenances of each, but they were quiet, peaceable and submissive. We observed two or three of them coughed frequently, and that in nearly all of them, one or both ears were suppurating, with glandular swellings on each side of the neck. Scrofulosis had most faithfully daguerreotyped itself in these children, and the pictures were by no means pleasant, but painful to contemplate. The parents, themselves, were so weak in intellect as not to perceive or know that their children were mentally defective. We looked upon these children with pity, as they were capable of feeling and suffering, and without the capacity of caring for themselves, and in the course of time would become objects of charity and public care, provided they lived to old age. But this was not to take place, for, doubtless, they did not survive puberty. They were susceptible of anguish and suffering, however, inflicted upon them too, by the unnatural union of unnatural persons. In a moral, social, or civil point of view, had they the right to enter into a connection which would result in throwing upon the world a family of *idiots,* to become a heavy charge to others, or beings to suffer without physical or intellectual enjoyment? We, for our part, are ready with an emphatic negative reply. We repeat again what has already been maintained in former numbers of this Journal, that there should be special statuary enactments in each State prohibit ing it. Because of the miscellaneous character of the Western people, coming as they do from the different States of the Union, and from almost every country in Europe, their physical and intellectual characteristics will, doubtless, be improved, while those remaining in the older States and countries will deteriorate in proportion as they continue to mingle blood with blood.

